# Current Evidence on the Use of Antifilarial Agents in the Management of Bancroftian Filariasis

**DOI:** 10.1155/2011/175941

**Published:** 2010-12-30

**Authors:** Sumadhya Deepika Fernando, Chaturaka Rodrigo, Senaka Rajapakse

**Affiliations:** ^1^Department of Parasitology, Faculty of Medicine, University of Colombo, Colombo 08, Sri Lanka; ^2^University Medical Unit, National Hospital of Sri Lanka, Colombo 08, Sri Lanka; ^3^Department of Clinical Medicine, Faculty of Medicine, University of Colombo, Colombo 08, Sri Lanka

## Abstract

Many trials have explored the efficacy of individual drugs and drug combinations to treat bancroftian filariasis. This narrative review summarizes the current evidence for drug management of bancroftian filariasis. Diethylcarbamazine (DEC) remains the prime antifilarial agent with a well-established microfilaricidal and some macrofilaricidal effects. Ivermectin (IVM) is highly microfilaricidal but minimally macrofilaricidal. The role of albendazole (ALB) in treatment regimens is not well established though the drug has a microfilaricidal effect. The combination of DEC+ALB has a better long-term impact than IVM+ALB. Recent trials have shown that doxycycline therapy against *Wolbachia*, an endosymbiotic bacterium of the parasite, is capable of reducing microfilaria rates and adult worm activity. Followup studies on mass drug administration (MDA) are yet to show a complete interruption of transmission, though the infection rates are reduced to a very low level.

## 1. Introduction

There are nine filarial nematodes causing disease in humans. According to the location of the parasite and the pathogenesis, the disease can be classified as lymphatic, subcutaneous, and serous cavity filariasis. Two filarial worms, namely, *Wuchereria bancrofti *and *Brugia malayi* cause lymphatic filariasis. The World Health Organization (WHO) considers lymphatic filariasis to be a global health problem affecting approximately 120 million people in over 80 countries [1]. One-third of affected individuals are from South Asia and another one third is from Africa [1]. One sixth of the world population is at risk of infection [1]. 

The adult *W. bancrofti* worms live within the human lymphatic system. They have a long life span of 4–6 years. Females are viviparous and release thousands of microfilaria into the blood stream of the host after mating. These are taken up by vector mosquitoes during feeding, and the parasite undergoes several moults within the intermediate host to become the L3 larva which is the infective stage. During a feed, this larva enters the human blood stream and migrates to the lymphatics where it moults to become an adult worm [2]. There is a range of clinical manifestations in bancroftian filariasis with asymptomatic microfilaremics being at one end of the spectrum. Symptomatic patients may have acute (lymphangitis, lymphadenitis), chronic (elephantiasis, lymphoedema, hydrocoele, chyluria), or atypical (funiculitis, mastitis) manifestations [3]. Some may suffer from tropical pulmonary eosinophilia (TPE) due to the immunological hyperresponsiveness to the parasite [4].

The disease burden of lymphatic filariasis is significant. Chronic disease causes serious disfiguration and incapacitation of the patient with resultant stigma and marginalization. It is a disease of the poor, and it significantly affects their ability to earn an income. Many chronically ill patients are nonproductive for the rest of their life and become a burden to family and society [1, 5, 6]. This review focuses on the drug treatment of lymphatic filariasis caused by *W. bancrofti.*

## 2. Search Strategy and Methods

A MEDLINE search was carried out for all articles with the key word “*Wuchereria bancrofti*” in any field. The search was restricted to articles published in English within the last 10 years (1999–2009), as they would contain more recent data. There were 659 abstracts in the original search with these restrictions. The software, Endnote X1.01 was used to filter articles. Bibliographies of cited literature were also searched. All abstracts were read through independently by the three authors, and relevant ones were identified for review of the full papers. Related papers were also included. Where the full paper was not available online or as hard copies, we contacted the authors and obtained the articles. Suitable data was available in 73 papers.

Sources were screened for a well-described methodology, accurate statistical analysis, and an adequate sample size where relevant. Coding was done by three reviewers independently blinded to each other. Interreviewer agreement for final review was 100%. Data sources included reviews published in core clinical journals, cohort studies, interventional studies, case control studies, cross-sectional analysis, and epidemiological data. We reviewed 64 (87.6%) full papers from a selected 73. A summary of the cited literature is shown in Tables 1 and 2. 

One of the main issues that arose in evaluating the efficacy of therapies for bancroftian filariasis was the differences in outcome measures of treatment used in different trials. Of these we identified the following key outcome measures: (a) microfilaricidal effect, (b) clearance of antigenaemia, (c) macrofilaricidal effect, and (d) prevention of clinical effects or complications of filariasis. The key pharmacological regimens in the management of lymphatic filariasis are, diethylcarbamazine (DEC), albendazole (ALB), and ivermectin (IVM) either used alone or in combination. We assessed the efficacy of each of these drugs or drug combinations in achieving the above-mentioned outcome measures. The value of these drugs in treatment of the individual and with regards to mass treatment, were considered separately.

## 3. Standard Treatment with DEC

DEC has been used to treat lymphatic filariasis for over 50 years. Its mechanism of action is still not fully understood. Earlier studies suggested that DEC had no direct effect on microfilaria as exposure to high concentrations of DEC left them unharmed [7]. Later, evidence from in vitro studies suggested that DEC blocks the cyclooxygenase pathway in parasites and leads to death of microfilaria [8]. Peixoto et al. [9] have demonstrated that DEC induces apoptosis in *W. bancrofti* microfilaria following exposure. Due to this microfilaricidal activity of DEC, the blood is cleared of microfilariae and the opportunity for mosquito borne transmission to occur is reduced. Further, filaria-associated haematuria and proteinuria are reversed. The macrofilaricidal action of DEC is not intended to reverse existing lymphatic damage but prevent further adult worm associated lymphatic damage and dysfunction [10]. The 12-day regimen of 72 mg/kg of DEC treatment remained the standard treatment for bancroftian filariasis for many years [11]. However, currently studies have indicated that single-dose treatment with 6 mg/kg DEC has comparable macrofilaricidal and long term microfilaricidal efficacy, and this has been discussed. The 12-day course of DEC provides more rapid short-term microfilarial suppression, but when other factors are considered, including cost, convenience, and patient compliance it seems feasible to recommend single-dose treatment for individual patients with *W. bancrofti* infection. Single-dose treatment can be repeated every 6–12 months for persons who remain infected. However the 12-day regimen which reduces microfilarial density more rapidly is recommended for patient with TPE or hematuria, both of which are associated with microfilariae rather than the adult worm [12]. DEC is not used in areas endemic for onchocerciasis due to an increased side effect profile [13, 14].

## 4. Evidence from Clinical Trials on Antifilarial Agents

### 4.1. Single-Dose Treatment

Single dose treatment with DEC is as effective as the older standard 12-day course of DEC, but has fewer adverse effects and results in enhanced population compliance and decreased delivery costs [15]. Single-dose therapy with DEC has been assessed in several trials (Table 1). In a prospective study in Egypt, a single dose of DEC achieved a microfilaria-clearance rate of 69% (*n* = 20) after 1 year while the reduction in antigenaemia was less satisfactory (*n* = 86, 40.7%) [16]. A prospective trial in Sri Lanka recorded a 74–80% reduction in microfilaria density (19–28% microfilaria-clearance rate) with a single dose of DEC 6 mg/Kg, 1 year after treatment [17]. However, the benefit of a single dose therapy may not be long lasting, as shown in a 10-year followup study in Orissa, India [18]. In this study of 44 patients, only 57% and 18% tested negative for microfilaria and antigenaemia, respectively, at the end of the followup period of 10 years after a single standard dose of DEC. Similar evidence comes from Freedman et al. [19] who demonstrated significant levels of antigenaemia (clearance rate of only 12%) at two years despite a more aggressive treatment regimen with DEC (repeated dosing with 6 mg/kg for 12 days at 0, 6, 12, 18 months). 

Pani et al. [20] demonstrated that either single dose administration of DEC, ALB, or combination therapy were not different from each other with regard to microfilaria-clearance rates and reducing antigenaemia (*P* > .05). Marked reduction in mean geometric parasite density (*P* < .05) as well as antigenaemia optical density (*P* < .01) was seen in all groups at followup in 1 year. 

Ivermectin is the third drug used in the treatment of bancroftian filariasis. Regarding monotherapy with IVM, Stolk et al. [21] demonstrated that single dose IVM alone can achieve a high microfilaria kill rate and a worm productivity loss at 1 year (96% and 82% on average, resp.). In comparison, the rates for the DEC treated group were very much lower (57% and 67%, resp.). Interestingly a similar trial by Reddy et al. [22] (with high-dose IVM) who followed up patients for two years suggests that both the tolerability and efficacy of the two drugs (IVM, DEC) were not significantly different between gender, age, and weight classes of patients at two years, although IVM showed a better response at one year. IVM is avoided in areas endemic for *Loa loa * [23, 24]. 

### 4.2. Single Dose versus Combination Therapy

There are several studies comparing single drug therapy with combination therapy. Dreyer et al. [25] report a significant reduction in macrofilaricidal effect in the combined regime of DEC and ALB compared to DEC alone (*P* = .016) with no additional effect on microfilaria reduction rates. In a large randomized controlled clinical trial, Bockarie et al. [26] demonstrated that single dose DEC (6 mg/Kg of body weight) has no superiority over combination therapy (DEC with ALB 400 mg single dose) in reducing microfilaria rates over a followup period of 2 years. Nonetheless, combination therapy had a significant macrofilaricidal effect (*P* < .003) compared to DEC alone at the end of followup (the antigen Og4C3 prevalence was used to measure adult worm activity). Fox et al. [27], in a large scale (*n* = 990) randomized placebo-controlled trial, showed that combination therapy has a significant microfilaricidal effect compared to DEC or ALB used alone (*P* < .03). In a smaller prospective study, Hussein et al. [28] (*n* = 58) demonstrated that ultrasonographic evidence of adult worm nests showed a 90% reduction after 1 year from start of combination therapy with DEC+ALB. It was also shown that single dose therapy versus multiple doses (over 7 days) had no additional benefit in this regard. Conflicting evidence comes from El Setouhy et al. [29] who report significantly greater microfilaricidal and macrofilaricidal effects at 1 year for multiple doses of combined therapy with ALB+DEC. 

IVM is usually administered in combination with ALB. Two studies have shown that the combination is more effective in killing microfilaria in humans and reducing infection rates in the vector than individual drugs or placebo [30, 31]. There is some speculation that IVM affects the reproductive capacity of female worms [32]. Five clinical trials in Sri Lanka [33, 34], Ghana [35, 36], and Tanzania [37] with a followup for 1-2 years have demonstrated the efficacy of ALB and IVM combination on microfilaria clearance. Two studies [33, 34] had an arm treated with high-dose IVM (400 *μ*g/Kg) and ALB (Table 1). The Sri Lankan trials also compared the efficacy of IVM and ALB with DEC and ALB. Almost all regimens with IVM demonstrated a rapid kill rate of microfilaria with higher doses showing a greater reduction in microfilaria rates. A subsequent mathematical-model-based analysis based on these 5 trials has shown that the reduction of microfilaria with DEC and ALB is slower but long lasting [38]. While constructing the model, the authors have tried to assess the trends in microfilaria densities in several trials after starting treatment with different antifilarial drug combinations. Since the study populations were from endemic areas, it was assumed that before the start of treatment the microfilarial densities were at an equilibrium (production matched by elimination) and the effect of drugs were described in two terms; microfilaria loss (fraction of microfilaria killed) and worm productivity loss (fraction of microfilaria permanently rendered incapable of reproducing). As the maximum followup was 2 years in the studies entered into the model, new infections were thought not to affect the equilibrium as they would not yield microfilaria during this period due to the long premature period of the worm. By using this model authors have also tried to estimate how the microfilarial densities would change in the posttreatment period. From observed data, DEC- and ALB- based trials had an almost 100% worm productivity loss at both high and low doses of ALB while only the high-dose combinations of IVM and ALB recorded similar results. Even after allowing for acquisition of new infections, the efficacy estimates did not vary between the trial arms. Ismail et al. [33] recommend that ALB and DEC are a better option for mass chemotherapy for endemic populations, based on the high rates of microfilarial clearance.

Bockarie et al. [39–41], in a prospective study, recruited nearly 2500 people to receive four rounds of annual treatment in Papua New Guinea. They were randomly assigned to two treatment groups to receive either DEC and IVM or DEC alone. After four rounds of treatment (77%–86% compliance rate), microfilaria positive infections were reduced by 86–98%. Chronic manifestations such as lymphoedema and hydrocoele were also significantly reduced in the population (*P* = .04, <.001, resp.). There was no difference in the two drug regimens with regard to efficacy. However, the combination of IVM and DEC rapidly reduced microfilaria positivity, especially in high-transmission areas. Still, at the end of the four years, the odds of microfilaria transmission were the same for both regimens.

A double-blind clinical trial on a head-to-head comparison of high-dose IVM and DEC showed that IVM has a higher microfilarial clearance and DEC has a higher antigenaemia clearance [42]. Both therapies had residual microfilaria and Ag levels comparable with each other following 1 and 3 months of dosing, respectively.

### 4.3. Treating the Masses: Evidence from Mass Treatment Programmes

In 1997, WHO drew the blueprint to eliminate lymphatic filariasis by 2020 [1]. Mass drug administration (MDA) in endemic areas/countries was considered to be more cost effective than detecting and treating infected individuals. The low side effect profile of drugs and the pledge by two pharmaceutical companies to provide them free of charge, as long as necessary, made MDA a good elimination strategy. Currently, an estimated 754 million people in 81 countries are targeted for MDA and 546 million are already receiving it. Sixty-one countries have completed mapping of endemic areas, and in another 16 it is in progress. China and South Korea have already declared the elimination of  lymphatic filariasis as a public health priority [43]. The use of MDA in filariasis gives the unique opportunity to see how the results of smaller clinical trials are valid when the drugs are administered to masses of general population. Currently there are three regimens approved for MDA, namely, DEC with ALB, IVM with ALB, and DEC- medicated salt [43]. 

In Burkina Faso, MDA with IVM alone (for onchocerciasis) has shown an indirect benefit by lowering *W. bancrofti* microfilaria rates. Kyelem et al. [44] reported that, in comparison to nonendemic and, therefore, nontreated communities, the treated communities had significantly lower microfilaria rates after six rounds of annual treatment. However, the rates of hydroceles and lymphoedema did not differ in the two communities. Furthermore, an entomological survey by Richards et al. [45] did not find significantly lower rates of infection in mosquitoes with *W. bancrofti* larvae in treated and untreated communities with IVM in Nigeria (MDA for onchocerciasis). All communities had good compliance with MDA, but only two rounds of treatment were completed in three of the five communities studied. A community-based trial on head to head comparison on the efficacy of DEC (6 mg/kg, single dose) and IVM (400 *μ*g/kg, single dose) in South India has shown that after 10 years of annual MDA, DEC had the potential to interrupt the transmission of filariasis while IVM was less able to do so [46]. 

ALB and DEC are used as a combination for MDA in many nononchocerciasis-endemic populations, and has been proven to be effective. After 6 years of MDA in American Samoa, the antigenaemia prevalence dropped from 11.5% in 2001 to 0.95% in 2006 (*P* < .0001) with this regimen [47]. MDA for five years with DEC alone in Fiji has also shown a statistically significant reduction in microfilaria rates irrespective of the pretreatment microfilaria rates [48].

### 4.4. The role of DEC-Fortified Salt

DEC-medicated cooking salt has been used to facilitate mass treatment and has proved to be very effective and safe. DEC fortified salt has been recommended mainly for control programmes chiefly because of its ability to clear microfilaraemias without causing adverse reactions. It is anticipated that this approach would ensure compliance. The lack of adverse effects is due to the very slow clearance of parasitaemia compared with that achieved with tablets. DEC medicated salt plays a major role in the Chinese filariasis control programme and proved successful in more limited trials in India, Brazil, and Tanzania [49–52]. It has been shown that DEC salt is more effective than single dose DEC in reducing the prevalence of microfilaraemia. DEC fortified salt may be useful in areas where the mobilisation of the population for annual drug distribution is difficult. Common salt medicated with 1–3 g of DEC per kg is used for atleast 6–12 months. It is well tolerated and safe to use in pregnancy. It is colourless, odourless, thermostable, and tastes the same as ordinary cooking salts. The macrofilaricidal effect of very low-dose DEC as used in the DEC medicated salt is not sure. Low dose DEC in salt minimizes or avoids completely the known side effects of treatment, including both acute pharmacologic effects of high doses and Mazzotti-like inflammatory reactions (probably due to dying microfilariae) induced by moderate and high doses [53].

Several pilot studies have been conducted using salt fortified with DEC in endemic communities in India, Tanzania, and Brazil. All of them have demonstrated effective microfilaria kill rates [49, 51, 52, 54–56]. A large community-based trial in Haiti, over a period of 1 year has shown that DEC- and Iodine-fortified salt lowered the prevalence and intensity of microfilaraemia by 95% [57]. However, the impact on adult worms was less (60% reduction in Og4C3 antigenaemia and a nonsignificant reduction in motility of worm nests detected by ultrasound).

## 5. Resurgence after MDA: Is Eradication Possible?

WHO aims to achieve cessation of transmission of infection after 4–6 rounds of therapy yearly (which corresponds to the fecundity of the adult worms) provided the compliance is good. However, initial small-scale trials failed to completely clear microfilaria rates with either combination of drugs, though the ALB+DEC combination had a lasting effect. The followup studies after several MDA rounds confirm this. Meyrowitsch et al. [58] report that after 10 years of MDA with DEC (given in three regimens) the microfilaria levels were reaching the pretreatment value in all communities. Many of the recurrences were in previously microfilaria positive individuals indicating the possibility of reproduction from surviving female adults. A three-arm community-based trial in India assessed the impact of two rounds of annual MDA after 3 years since the last dosing. The improvement with MDA was sustained when therapy was combined with vector control [59, 60]. The importance of vector control and understanding of local transmission dynamics are also underscored by Simonsen et al. [61], who have shown that after two rounds of MDA, mosquitoes carrying infective larvae were not reduced, though mf rates in the community were significantly less. The most suitable cohort to study the impact of long-term MDA is the Maupiti cohort of French Polynesia where semiannual MDA has been combined with vector control since 1955. Two surveys in 1985 and 1989 showed a 0% microfilaria rate which gave hope that eradication was complete. Nonetheless, Esterre et al. [62] in two repeated cross-sectional analyses in 1997 and 1999 have shown residual microfilaraemia and antigenaemia (0.4% and 4.6%, resp.) with a 1.4% infectivity rate in vector population. There are several plausible explanations for this observation: efficiency of the vector, resistance to DEC, and prolonged longevity of adult worms. These findings cast doubt on the possibility of a complete “eradication” of filariasis with MDA. 

In this background, Micheal et al. [63, 64] suggest that plans to control lymphatic filariasis should be more pragmatic, flexible, economically sensitive, and sequential. They suggest that the first target in an elimination programme should be to achieve an infection rate at which chronic manifestations of infection (causing more productivity loss and DALYs) become negligible despite ongoing infection. Using a mathematical model based on available data it is suggested that a microfilaria rate of 3.55% at a blood sampling volume of 1 ml will achieve this. This target is both achievable and sustainable with current MDA regimens.

## 6. Resistance to Drugs

One factor linked to resurgence of infection following MDA is the resistance to drugs. It is impossible to assess the resistance to DEC as its mechanism of action is still obscure. However, resistance to IVM and ALB has been reported in nematodes in veterinary practice. In 2004, resistance to IVM was reported in the human parasite *Oncocerca volvulus *[65]. There are yet no confirmed reports of resistance in *W. bancrofti* for IVM. 

The main cause for concern, however, is resistance to Benzimidazoles (BZ), namely, ALB. The resistance to BZs (ALB, Mebendazole) is seen in many nematode parasites due to single nucleotide polymorphisms (SNP) [66]. Two SNPs substituting tyrosine for phenylalanine of the *β* tubulin protein of nematodes confer resistance to ALB in veterinary practice. Schwab et al. [67, 68] has demonstrated that similar SNPs exist in *W. bancrofti* in untreated populations, and such mutations are selected for after mass treatment. The impact of this may not be felt immediately in the population as microfilarial rates drop rapidly with combined chemotherapy. Still, if resurgence occurs in future, resistant genotypes with a selection advantage may predominate in the parasite population making ALB resistance a significant problem. However, as some authors point out, the real problem is not related to *W. bancrofti* at all it is the possibility of other intestinal nematodes developing resistance to BZs due to large scale exposure to ALB during MDA that could pose a serious threat to health of children and adults in endemic areas [69].

## 7. The Place for Targeting Wolbachia with Doxycycline in Treatment Regimens

*Wolbachia *is an intracellular symbiotic bacterium of filarial parasites. It plays an essential role in larval moulting, adult worm survival, and female worm fertility. Killing the bacterium with doxycycline has shown promise in many studies by reducing adult worm activity [70, 71]. Though doxycycline therapy has been experimented with for treating infections with other filarial worms, the first trial with regard to *W. Bancrofti* was conducted in 2005 by Taylor et al. [72] after 8 weeks of doxycycline 200 mg/d, microfilaraemia was almost eliminated (*P* < .001), antigenaemia was halved (*P* = .015), and ultrasonographically demonstrated adult worm activity was significantly less (*P* < .0001) in the treatment group versus placebo group (after 14 months of followup). There were no serious side effects with treatment. Subsequent studies with shorter courses of doxycycline (6, and 4 weeks, resp.) have shown a similar effect. In these studies antibacterial therapy was followed up with IVM+ALB combined therapy [73, 74]. However, a 3-week course of the drug failed to show an adequate macrofilaricidal effect [75]. 

In addition to killing the endosymbionts and reducing the filarial worm load, doxycycline also improves clinical manifestations of filariasis. The levels of vascular endothelial growth factor C (VEGF-C) and soluble vascular endothelial growth factor receptor-3 [(s)VEGFR-3], which has been shown to be important in pathogenesis of filariasis in animal models, were lowered in test subjects following doxycycline therapy [76]. The macrofilaricidal effect of doxycycline is slow compared to DEC, and the side effects seen after DEC treatment (abscesses, etc.) are not seen. Addition of doxycycline to treatment regimens will have a beneficial effect especially in Onchocercaria endemic areas where DEC is contraindicated. IVM used in these areas have no or minimum macrofilaricidal effect.

## 8. Limitations

This review was limited to articles published in English within 1999–2009 time period. While attempts were made to search related literature as well, it is possible that important studies published in other languages and outside the search limits were missed.

## 9. Conclusions

WHO has outlined two objectives for its campaign of MDA: to interrupt transmission and to reduce morbidity of disease [1]. The best combination of drugs for an MDA programme was still not clarified by the time the programmes were launched in endemic areas. Clearly, one of the main difficulties in determining the efficacy of individual drugs is that different endpoints have been used in different trials (microfilaria-clearance rates, antigenaemia-clearance rates etc.), and correlating efficacy based on these endpoints and actual clinical efficacy is difficult. As individual drugs, IVM reduced the microfilaria rates rapidly, but DEC had more macrofilaricidal effects with a higher clearance of antigenaemia. The only available large-scale community-based trial to evaluate IVM versus DEC, showed that the latter was more effective in interrupting transmission [46]. The evidence for benefits of combination therapy is also conflicting but many studies favour it. Only two studies quoted above show no difference between single and combination therapy while Dreyer et al. [25] actually report a loss in macrofilaricidal effect of DEC when given in combination. However, this study uses ultrasound evidence to assess outcome rather than the antigen clearance. It may be difficult to correlate antigenaemia to macrofilaricidal effects as shown by a large scale study in Sri Lanka. After a 12-day course of DEC, 78% showed microfilaria clearance. However, of 76% of those “cured” parasitologically were still positive for the Og4C3 antigen at 17 months [77]. The ALB+DEC regimen was considered a better option for nononchocercaria endemic areas than the ALB+IVM regimen. Nonetheless, large-scale randomized clinical trials are not available to formulate evidence-based guidelines for chemotherapy, and currently only recommendations can be made in treating bancroftian filariasis based on available evidence. 

Despite 50 years of research into filariasis control, still many questions remain unanswered. These include basic issues like mechanism of action of DEC, best combination of drugs for elimination strategies, and evidence-based recommendations to treat lymphatic filariasis. Differences in the end-points of treatment studied add confusion to the benefits of the different drugs and drug combinations. Much of the recommendations for therapy are based on microfilaraemia and antigenaemic clearance; evidence of reduction of clinical manifestations has not been studied adequately in either large-scale population surveys or clinical studies. The need to identify clear endpoints in future clinical trials and population surveys cannot be overemphasised. The policies of MDA also need to be reviewed, and, as community-based studies have shown, despite intensive therapy, that infection rates have not been reduced to zero. It is important to combine vector control with MDA and develop elimination strategies that are flexible and achievable in local context. Perhaps it is more important to target an infection rate that reduces the impact of lymphatic filariasis as a public health problem rather than aim towards total eradication, as eventually what matters is that the clinical manifestations of lymphatic filariasis are prevented.

## Figures and Tables

**Table 1:** Summary of clinical trials on drug treatment quoted in text.

Authors	Year	Study design	Drug doses	Outcome
Bockarie et al.	2007	Randomized controlled clinical trial	Single-dose DEC at 6 mg/kg versus DEC plus ALB 400 mg single dose	No difference in microfilaricidal effect but combination therapy had more macrofilaricidal effect.
Fox et al.	2005	Randomized placebo- controlled trial four arms	(i) DEC 6 mg/kg single dose (ii) ALB 400 mg single dose (iii) Combination of both (iv) Placebo	Combination therapy has a significant microfilaricidal effect than either DEC or ALB used alone.
Hussein et al.	2004	Prospective study two arms	(i) DEC 6 mg/kg and ALB 400 mg single dose (ii) Same repeated daily for 7 days	Combination therapy reduced adult worm activity by 90% after 1 year. No benefit of multiple dosing versus single dosing beyond 3 months.
El Setouhy et al.	2004	Randomized clinical trial two arms	(i) DEC 6 mg/kg and ALB 400 mg single dose (ii) Same repeated daily for 7 days	Greater and significant microfilaricidal effects 1 year after treatment (effect on adult worms were similar) for multiple dose combined therapy.
Pani et al.	2002	Double-blind hospital based clinical trial three arms	(i) DEC 6 mg/kg single dose (ii) ALB 400 mg single dose (iii) Combination of both	Single dose administration of DEC, ALB, or combination therapy were not different from each other with regard to microfilaria-clearance rates and reducing antigenaemia.
Dreyer et al.	2006	Randomized controlled clinical trial two arms	(i) DEC 6 mg/kg single dose (ii) DEC 6 mg/kg + ALB 400 mg single dose	Significant reduction in macrofilaricidal effect in the combined regime compared to DEC alone (*P* = .016) with no additional effect on microfilaria rates.
Ramzy et al.	2002	Prospective study	Single-dose DEC 6 mg/kg	DEC single dose therapy achieved a microfilaria-clearance rate of 69% in one year with a 40.7% reduction in antigenaemia.
Weerasooriya et al.	1998	Prospective study	Single-dose DEC 6 mg/kg	A reduction in microfilaria density by 74–80% and a 19–28% microfilaria clearance rate at 1 year after treatment.
Weerasooriya et al.	2002	Prospective study	A 12-day course of DEC 6 mg/kg	Microfilaria clearance achieved in 78% of infected people. However, 76.1% of them remained positive for the Og4C3 antigen at end of 17 months.
Beuria et al.	2002	Prospective study	DEC 6 mg/kg for 12 days	Only 57% and 18% tested negative for microfilaria and antigenaemia, respectively at the end of the followup period of 10 years.
Freedman et al.	2001	Prospective study	DEC 6 mg/kg for 12 days at 0,6,12,18 months	Only 12% clearance rate of antigenaemia at the end of a followup period of 2 years.
Beach et al.	1999	Randomized placebo-controlled clinical trial four arms	(i) IVM 200–400 *μ*g/kg single dose (ii) ALB 400 mg single dose (iii) Combination of both (iv) Placebo	Combined therapy with ALB and IVM reduces microfilaraemia more than placebo or individual drugs
Richards et al.	2005	Prospective entomological survey		The combination of ALB and IVM appears to be superior to IVM alone for reducing the frequency of *W. bancrofti* infection in mosquitoes.
Dunyo et al.	2000	Double-blind placebo-controlled field trial two arms	(i) IVM 150–200 *μ*g/kg single dose (ii) IVM 150–200 *μ*g/kg + ALB 400 mg single dose	Both IVM and combination treatment appeared effective for control of *W. bancrofti* infections, but the difference in efficacy between the 2 treatments after 12 months appeared to be minimal.
Ismail et al.	1996	Double-blind clinical trial two arms	(i) 400 *μ*g/kg of IVM 12 fortnightly doses (ii) 10 mg/kg of DEC 12 fortnightly doses	IVM has higher microfilarial (mf) clearance, and DEC has higher antigenaemia (ag) clearance. Both therapies had residual mf and ag levels comparable with each other following 1 and 3 months of dosing, respectively.
Ismail et al.	1998	Blinded four-arm clinical trial	(i) ALB 600 mg single dose (ii) ALB 600 mg + IVM 400 *μ*g/kg (iii) ALB 600 mg + DEC 6 mg/kg (iv) IVM 400 *μ*g/kg + DEC 6 mg/kg	All 4 treatments significantly reduced mf counts, but ALB/IVM was the most effective regimen for clearing mf from night blood. All 4 treatments had significant activity against adult *W. bancrofti* with DEC+ALB having the greatest effect (Followup:15 months).
Ismail et al.	2001	Blinded three-arm clinical trial	(i) ALB 400 mg + IVM 200 *μ*g/kg (ii) ALB 400 mg + DEC 6 mg/kg (iii) ALB 600 mg + IVM 400 *μ*g/kg	All 3 treatments significantly reduced mf counts, with the ALB-DEC-treated group showing the lowest mf levels at 18 and 24 months after-treatment. All 3 treatments had significant activity against adult *W. bancrofti*; ALB-DEC combination had the greatest activity.
Makunde et al.	2003	Crossover, double-blind design two groups	For group with coinfection with *W. bancrofti* and *O. volvulus*-single dose of IVM 150 *μ*g/kg + 400 mg ALB versus placebo. Treatment was crossed over after 5 days of initial dosing For group with only *W. bancrofti* infection-Single dose of ALB 400 mg versus ALB+IVM 150 *μ*g/kg	There was no significant difference in the reduction of microfilaraemia following treatment with ALB and IVM in groups with single or coinfection. IVM plus ALB is a safe and tolerable treatment for coinfection of bancroftian filariasis and onchocerciasis.
Stolk et al.	2005	Prospective two-arm study two arms	(i) 400 *μ*g/kg IVM single dose (ii) 6 mg/kg DEC single dose	IVM on average killed 96% of Mf and reduced Mf production by 82%. DEC killed 57% of Mf and reduced Mf production by 67%.
Reddy et al.	2000	Double-blind two-arm clinical trial	(i) 400 *μ*g/kg IVM single dose (ii) 6 mg/kg DEC single dose	Tolerability and efficacy of the two drugs (IVM, DEC) were not significantly different between gender, age, and weight classes of patients at two years.
Debra et al.	2006	Double-blind placebo-controlled trial	Doxycycline 200 mg/d for 6 weeks followed by IVM 150 *μ*g/kg + 400 mg ALB single dose 4 months later	*Wolbachia* load, microfilaraemia, antigenaemia, and frequency of filarial dance sign were significantly reduced in microfilaraemic patients up to 24 months in the doxycycline group compared to the placebo group.
Debra et al.	2009	Double-blind placebo-controlled trial	Doxycycline 200 mg/d for 6 weeks followed by IVM 150 *μ*g/kg + 400 mg ALB single dose 4 months later	Six-week regimen of doxycycline treatment showed improvement of clinical features of hydrocoele patients with active infection.
Taylor et al.	2005	Double-blind placebo-controlled randomized trial	Doxycycline 200 mg/d for 8 weeks	An 8-week course of doxycycline is a safe and well-tolerated treatment for lymphatic filariasis with significant activity against adult worms and microfilaraemia.

**Table 2:** Summary of followup studies on cohorts receiving mass drug administration.

Author	Year published	Design	Drug regimen	Followup	Conclusions
Bockarie et al., Papua New Guinea	2002	Prospective controlled randomized clinical trial	(i) DEC 6 mg/kg single dose (ii) DEC 6 mg/kg + IVM single dose	5 years	Microfilaria positive infections were reduced by 86%–98%. Chronic manifestations such as lymphoedema and hydrocoele were also significantly reduced in the population. No difference in two regimens at end of followup.
Kyelem et al., Burkina faso	2003	Prospective two-arm study	Communities receiving IVM 150 *μ*g/kg annually compared with communities not receiving MDA	6 years	Long-term IVM (given for onchocerciasis) significantly reduced *W. bancrofti* and *M. perstans *microfilaraemia.
Richards et al., Nigeria	2005	Cross-sectional entomological survey	Communities receiving IVM 150 *μ*g/kg annually	2-3 annual rounds of chemotherapy completed	Annual therapy with IVM for onchocerciasis has not interrupted transmission of *Wuchereria bancrofti. *
Ramaiah et al.,	2007	Community-based followup study with two arms	DEC 6 mg/kg, single dose annual therapy versus IVM 400 *μ*g/kg single dose annual therapy	10 years	DEC had the potential to interrupt transmission while the capability of IVM to do so was less.
Liang et al.,	2008	Followup study	DEC + ALB standard dosing	6 years	The antigenaemia prevalence dropped from 11.5% in 2001 to 0.95% in 2006 (*P* < .0001).
Mataika et al., Fiji	1995	Followup study	Annual single dosing of DEC 6 mg/kg	5 years	MDA with DEC alone led to a statistically significant reduction in microfilaria rates irrespective of the pretreatment mf rates.
Freeman et al., Haiti	2001	community-based trial	DEC medicated salt	1 year	DEC and Iodine fortified salt lowered the prevalence and intensity of microfilaraemia by 95%. Impact on adult worms was less.
Meyrowitsch et al., Tanzania	1996	community-based trial	Comparison of four strategies of community treatment with DEC 6 mg/kg (i) 12 day regimen (ii) Semiannual single dose treatment (iii) Monthly low dose regimen (iv) DEC medicated salt	2 years	Strategies III and IV were equally effective, and superior in clearing microfilaraemias and in reducing mf geometric mean intensities compared to strategies I and II.
Meyrowitsch et al., Tanzania	2004	community-based trial	Followup of above-study	10 years	Microfilaria rates were reaching pretreatment values in all communities.
Fan et al., China	1990	Community-based trial	DEC medicated salt	12 years	Microfilaria rates and infection rates were reduced from 9.6% to 0.3% and 9.1% to 0.8%, respectively.
Liu et al., China	1992	Community-based trial	DEC medicated salt	4 years	Microfilaria rates dropped from a range of 1.56–11.81% to 0.05% in the communities studied.
Sunish et al., India	2002	Community-based trial with three arms	Group A: MDA with annual single dose of IVM 400 *μ*g/kg + DEC 6 mg/kg Group B: MDA with vector control Group C-Placebo	3-4 years	The improvement with MDA was sustained in the second group while resurgence occurred in the first group.
Simonsen et al., Eastern Africa	2004	Community-based trial in high-endemicity and low-endemicity communities	Semiannual treatment with DEC 6 mg/kg	1 year	Transmission rates dropped only in high endemicity communities, but it cannot be entirely attributed to MDA.
Esterre et al.,	2001	Community-based followup study	Semiannual treatment with DEC 6 mg/kg for more than 30 years	34 years	Microfilaria and antigenaemia rates were very low but not zero.
